# Serum amylase activity altered by the ABO blood group system in Chinese subjects

**DOI:** 10.1002/jcla.22883

**Published:** 2019-04-02

**Authors:** You‐Fan Peng, Hemant Goyal, Hao Lin, De‐Chen Liu, Ling Li

**Affiliations:** ^1^ Department of Endocrinology, School of Medicine, Zhongda Hospital Southeast University Nanjing China; ^2^ Pancreatic Research Institute Southeast University Nanjing China; ^3^ Department of Internal Medicine, School of Medicine Mercer University Macon Georgia USA; ^4^ Department of Clinical Science and Research, School of Medicine, Zhongda Hospital Southeast University Nanjing China

**Keywords:** ABO blood type, Chinese subjects, Serum amylase

## Abstract

**Objective:**

The underlying interactions between ABO blood group antigens and pancreatic exocrine tissue have been demonstrated, and serum amylase was synthesized by pancreatic ductal cells. Thus, we investigated the link between ABO blood type and serum amylase activity in Chinese subjects.

**Methods:**

Our study included 343 relatively healthy Chinese individuals, and the data were retrieved from electronic medical record database.

**Results:**

A increased trend was observed for serum amylase activity in ABO blood type distribution, and we found that serum amylase activity was remarkable increased in subjects with O blood type compared to those with non‐O blood type (*P* = 0.013). Logistic regression analysis indicated that serum amylase was independently associated with individuals with O blood group (adjusted odds ratio 1.574; 95% CI, 1.022‐2.425, *P = *0.039).

**Conclusions:**

The present evidence suggests a significant link between serum amylase activity and ABO blood type in the study population, indicating ABO blood type may be associated with the susceptibility of pancreatic disease.

## 1 INTRODUCTION

Amylase is an exocrine enzyme secreted by pancreas and salivary glands, which contributes to convert starch and glucose into smaller components.^1^ Under normal physiological conditions, approximately 40%‐45% serum amylase derives from the pancreas, and 55%‐60% are released from salivary glands.^1^ In clinical practice, serum amylase has been used to provide the diagnostic support for acute and chronic pancreatitis. It is reported that serum amylase activity is related with gestational diabetes mellitus.^2^ Moreover, serum amylase activity is linked to the islet β cell function in type 2 diabetes patients.^3^ In fact, serum amylase has been regarded as a biochemical marker to assess pancreatic exocrine function in patients with diabetes mellitus.^4^

The ABO blood type antigens (A, B, and H determinants) are glycoproteins presenting human blood types and are widely distributed on red blood cell surface and normal tissue throughout the body.^5^ Several studies have found that inherited blood group antigens were associated with disease risk such as cardiovascular disease, thrombotic vascular disease and stroke.^6^, ^7^, ^8^ In addition, the risk relationship between ABO blood type and pancreatic carcinoma has been suggested,^9^ and the incompatible expression of blood type antigens has been observed in pancreatic cancer cells, which indicates the expression of Lewis antigen in pancreatic cancer.^10^ Importantly, ABO blood group has been found to be associated with population‐wide risk of chronic pancreatitis.^11^ It is not difficult discover that these studies may suggest the underlying interactions between ABO blood type antigens and pancreatic disease. Thus, we investigate the link between ABO blood type and serum amylase activity in Chinese subjects.

## 2 MATERIALS AND METHODS

### 2.1 Study population

The retrospective study included consecutively 343 relatively healthy Chinese individuals, and the demographic and laboratory data were retrieved from electronic medical record database. Exclusion criteria: coronary heart disease, diabetes mellitus, hypertension, active infection, hyperuricemia, pancreatic disease, salivary gland disease, liver and kidney impairment, malignant tumor and mental disease. The present study was consented by the local ethical committee and was performed in line accordance with the Helsinki Declaration.

### 2.2 Laboratory tests

Serum samples were collected from all subjects with fasting state. Routine biochemical tests were carried out on the whole study population, including alanine aminotransferase (ALT), aspartate aminotransferase (AST), creatinine (Cr) and amylase. The ABO blood type was divided into A, B, O, and AB type in line accordance with the blood type test results in clinical laboratory.

### 2.3 Statistical analysis

Accumulated demographical and laboratory data were reported as proportion and median and interquartile (IQR). For the statistical purposes, the chi‐square test, Student's *t* test, and one‐way ANOVA were employed to compare the differences among groups appropriately, and non‐parametric statistical tests (ie, the Mann–Whitney *U* and Kruskal–Wallis *H* tests) were also used. Logistic regression analysis was performed to analyze the relation between serum amylase and ABO blood type. The statistical analysis was implemented by SPSS version 16.0 software. The level of statistical significance was set at *P*‐value < 0.05.

## 3 RESULTS

Table 1 indicates the main results grouped by ABO blood type. There were no significant statistical differences in sex, age, Cr, ALT and AST, a increased trend was observed for serum amylase activity in ABO blood type distribution, although there was no statistical difference.

**Table 1:** Demographic characteristics and biochemical parameters stratified by ABO blood type in the study population

Items	O	A	B	AB	*P*‐value
Sex (female /male)	68/78	32/41	43/54	13/14	0.964
Age(yr)	34 (20‐47)	37 (16‐47)	31 (17‐45)	37 (23‐48)	0.568
Alanine aminotransferase (U/L)	15 (11‐21)	17 (13‐22)	16 (13‐21)	18 (13‐25)	0.308
Aspartate aminotransferase (U/L)	20 (17‐23)	20 (17‐22)	19 (16‐22)	22 (18‐25)	0.159
Creatinine (umol/L)	66 (52‐77)	64 (53‐78)	65 (52‐79)	62 (54‐84)	0.966
Amylase (U/L)	73 (60‐88)	67 (55‐81)	68 (53‐85)	61 (45‐78)	0.053

**Figure 1:**
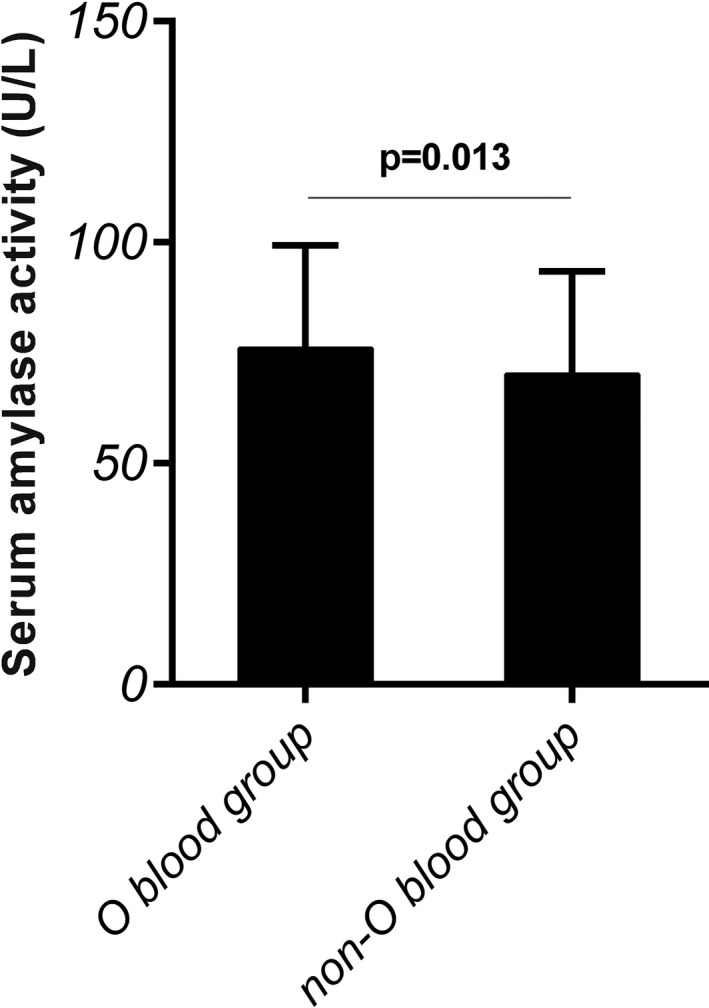
A significant difference was observed for serum amylase activity in O blood group and non‐O blood group distribution

We divided whole population into O‐type and non‐O‐type blood group. We found that serum amylase activity was remarkable increased in subjects with O blood type compared to those with non‐O blood type (*P* = 0.013), as shown as in Figure 1. No significant statistical differences were found between the two groups for other parameters.

Since the ABO blood type might affect serum amylase activity in the study, so whole population was separated according to the median of serum amylase activity, further, serum amylase was included as dependent variable in logistic regression analysis, subjects with O blood group and non‐O blood group, as well as age and sex were considered as independent variable in the model. The results indicated that serum amylase was independently associated with individuals with O blood group (adjusted odds ratio 1.574; 95% CI, 1.022‐2.425, *P = *0.039; Table 2).

**Table 2:** Logistic regression analysis between O blood group and non‐O blood group

Items	*P*‐value	OR	95% CI
Sex	0.476	0.855	0.556‐1.316
Age	0.332	1.006	0.994‐1.019
ABO blood group	0.039	1.574	1.022‐2.425

The serum amylase activity was divided according to the median, and was included as dependent variable into the logistic regression model.

## 4 DISCUSSION

The relationship between ABO blood type and serum amylase activity was investigated in the present population, and we found that subjects with O‐type group had an increased serum amylase activity compared to those with non‐O blood group independent of age and sex. In previous studies, the research hotspot for ABO blood type antigens has been focused on transfusion and organ transplantation. Recently, there were accumulated literatures associating ABO blood group with various diseases, such as cancer, infectious disease and cardiovascular disease.^12^, ^13^ Indeed, ABO locus single nucleotide polymorphism has been suggested to be linked with ischemic stroke.^8^ The relations of blood type A with increased risk of acute respiratory distress syndrome were found in patients with major trauma and severe sepsis.^14^ Furthermore, the links between human blood type and cancer have been reported in various studies, including pancreatic cancer.^9^, ^15^, ^16^ There were also evidences for the relationship between blood group and pancreatic disease.^9^, ^11^ Nakajima K et al 17 suggested a higher serum amylase activity in O blood type relative to A blood type in a general Japanese population; however, no study investigated the relationship between serum amylase activity and ABO blood type in Chinese people. Our study found that increased serum amylase activity was associated with O blood type relative to non‐O blood group in multivariate regression analysis. For the phenomenon, a possible mechanism is that the genetic linkage between blood group system and serum amylase in human pancreas. It is well demonstrated that human pancreatic amylase had genetic variation and polymorphism.^18^ There are two loci for four diverse isoamylases with codominant alleles, of which *amylase*_2 _controls pancreatic amylase secretion, and *amylase*_2_ locus is on chromosome 1 (*UN‐1*), interesting, the *amylase*_2_ locus is linked to Duffy blood group with gene order *UN‐1‐ amylase*_2_ *‐ Duffy* or *UN‐1‐ Duffy‐ amylase*_2_.^19^ Similarly, the linked alpha‐amylase *amylase*_2 _PKU locus is also associated with Duffy blood group.^20^ Besides, an early study found chromosomal loci had identical or closely link between red‐cell blood group antigen Ib and serum amylase variant.^21^ It can hence be hypothesized that the gene expression associated blood group antigens may control partly the secretion of pancreatic amylase due to gene linkage. Of course, other mechanisms may also contribute to explain the observed phenomenon.

Our study had several inevitable limitations. First, our current study was a small sample size in single medical center. Second, serum amylase might also derive from other organizations such as salivary glands, duodenum and lungs, and these organizations could express ABO blood group antigens, so the gene linkages between ABO blood type antigens and serum amylase might be not just in pancreatic tissue. Third, the relationship between serum amylase isozyme and ABO blood group was not identified. Finally, the study did not include other factors correlating the serum amylase activity, such as exercise, ethanol intake, and life‐style.

Additional study is needed to confirm the current results, in the current study, healthy individuals with O blood type maintain elevated serum amylase activity, and the fascinating relationship should be born and natural. Clinically, the variation of serum amylase is closely related to pancreatic disease such as pancreatitis, pancreatic exocrine dysfunction and pancreatic tumor.^22^, ^23^, ^24^ The present evidence suggests a significant link between serum amylase activity and ABO blood type in Chinese subjects, the important implication of our findings is that ABO blood type may be associated with the susceptibility of pancreatic disease.

## CONFLICT OF INTEREST

There were no financial conflicts of interest in all authors, and all authors participated sufficiently in the work to take public responsibility for appropriate portions of the content.
